# Impact of Socioeconomic Status on Post-Fall Recovery in Older Adults: A 12-Month Follow-Up

**DOI:** 10.1093/geroni/igaf122.2634

**Published:** 2025-12-31

**Authors:** Elisa-Marie Speckmann, Tania Zieschang, Tim Stuckenschneider

**Affiliations:** The Carl von Ossietzky University of Oldenburg’s School of Medicine and Health Sciences, Oldenburg, Niedersachsen, Germany; The Carl von Ossietzky University of Oldenburg’s School of Medicine and Health Sciences, Oldenburg, Niedersachsen, Germany; The Carl von Ossietzky University of Oldenburg’s School of Medicine and Health Sciences, Oldenburg, Niedersachsen, Germany

## Abstract

Falls in older adults can be a significantly disruptive event with long-term consequences. Given that socioeconomic status may influence health outcomes in this population, assessing its potential impact on post-fall recovery is essential. Therefore, we conducted a longitudinal study with data collection at three timepoints: four weeks, six months, and twelve months after a fall to assess the association of income and education with the progression of health outcomes. Data was obtained from the SeFallED study, which follows up on older adults, who were 60 years or older, attended the emergency department due to a fall, and were not admitted to the hospital. We calculated generalized least square models with first-order autoregressions to analyze the associations of education and income with physical performance, cognitive function, activities of daily living, life-space, and health-related quality of life. Age and sex were added as covariates. Over 12 months, we followed up with 267 individuals (mean age of 74.4±9 years, education: 10.2±1.9 years in school; income €2277.6±1806.3). Education was linked with physical performance (B = 0.25, p < 0.05), cognitive function (B = 0.70, p < 0.05), activities of daily living (B=-0.42, p < 0.05), and life-space (B = 2.59, p < 0.05). No further significant effects were found for either education or income. These findings show the need to account for disparities in education when designing secondary prevention programs for older adults. While education and income reflect socioeconomic differences, factors such as the subjective social status may offer additional insights and should be explored in future studies.

